# Metastasizing Malignant Granular Cell Tumor (Abrikossoff Tumor) of the Anterior Abdominal Wall, with Prolonged Survival

**DOI:** 10.1155/2019/9576487

**Published:** 2019-04-07

**Authors:** Yara A. Alnashwan, Khaled A. H. Ali, Samir S. Amr

**Affiliations:** ^1^Department of Pathology, Faculty of Medicine, Imam Abdulrahman Bin Faisal University, Dammam, Saudi Arabia; ^2^Department of Pathology, Almana General Hospital, Dammam, Saudi Arabia; ^3^Department of Pathology and Laboratory Medicine, King Fahad Specialist Hospital, Dammam, Saudi Arabia

## Abstract

Malignant granular cell tumor (MGCT) is a rare high-grade mesenchymal tumor of Schwann cell origin. MGCTs commonly affect thigh, extremity, and trunk; however, involvement of the abdominal wall is quite rare. It has poor prognosis with 39% mortality rate in 3-year interval. We report a 50-year-old female who had MGCT arising in the anterior abdominal wall and developed massive metastatic deposits in both lungs and in the right inguinal lymph nodes, with prolonged survival for 11 years. A brief review of the literature is presented.

## 1. Introduction

Granular cell tumors (GCTs), previously known as granular cell myoblastoma, are uncommon mesenchymal neoplasms of Schwann cell origin, composed of cells with granular eosinophilic cytoplasm, usually presenting as asymptomatic nodule, affecting adults, with women affected twice as men [1]. They occur in the dermis or subcutaneous tissues [2], oral mucosa including tongue [2, 3], gum (congenital epulis) [4], breast [5], vulva [6], and gastrointestinal and biliary tracts [7, 8]. In 5% of the reported cases, the tumor was multiple [2].

On the other hand, malignant granular cell tumors (MGCTs) are exceedingly rare high-grade malignant mesenchymal neoplasms, representing only 1%-2% of all GCTs [9]. In a study of 6,412 soft tissue sarcomas, 26 cases (0.4%) were diagnosed as MGCT [10], with 157 cases in various anatomical sites on record, documented in a review in 2018 [11]. When the tumor has malignant cytological features or when a benign-appearing tumor causes metastasis or death, it is considered malignant.

Most MGCTs commonly affect lower extremities, especially thighs and the trunk; however, involvement of the subcutaneous tissue of the abdominal wall is exceedingly rare [12].

 Herein, we report a case of MGCT arising in the anterior abdominal wall, who underwent surgical excision with wide resection margin and received chemotherapy, but developed massive metastases in the lungs and the right inguinal lymph nodes, with prolonged survival for 11 years, with a review of the relevant literature.

## 2. Case Report

A 50-year-old female presented in July 2007 to a peripheral general hospital with progressively enlarging mass in the anterior abdominal wall of one-year duration. On physical examination, she was well built with normal vital signs. A 7 x 6 cm firm irregular subcutaneous mass was felt in the right lower para-umbilical area of the anterior abdominal wall. On palpation, the abdomen was soft and lax with no intra-abdominal masses. There were no palpable lymph nodes. Her cardiac and respiratory examinations were unremarkable. Computerized Tomography (CT) scan of the abdomen revealed a mass within the abdominal wall. Her laboratory investigations were within normal limits. A plain chest X-ray was unremarkable.

Wide local excision with safety margins of the tumor was done. The postoperative period was uneventful, and she was discharged on the second postoperative day in a satisfactory condition.

Gross examination of the specimen showed an ellipse of skin that measured 13 x 10 cm with subcutaneous fatty tissue measuring 11 cm in thickness. On cut section, a well-defined nonencapsulated grayish yellow mass measuring 7 x 6 cm with foci of necrosis was seen. No skin infiltration or abdominal skeletal muscle involvement was noted.

**Figure 1:**
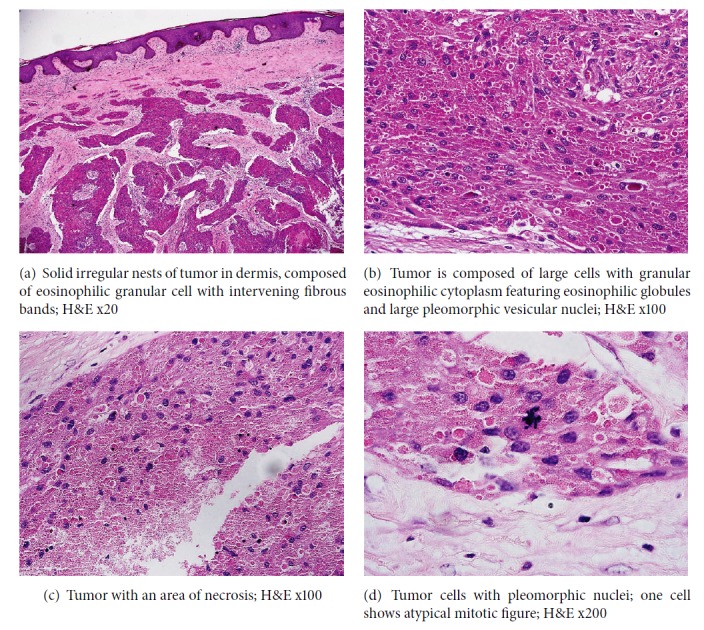


Microscopic examination revealed well-defined but nonencapsulated subcutaneous mesenchymal neoplasm composed of irregular islands of large cells with mostly round to oval pleomorphic vesicular nuclei, with markedly granular cytoplasm, and several large eosinophilic globules (Figure 1(a)). The nuclear cytoplasmic ratio was variable, with several cells having large nuclei. The tumor nests were surrounded by bands of fibrous connective tissue (Figure 1(b)).

Many foci of tumor necrosis and scattered mitotic figures, 4 per 10 HPF at a magnification of 200, were identified (Figures 1(c) and 1(d)). Margins of resection were free of tumor.

**Figure 2:**
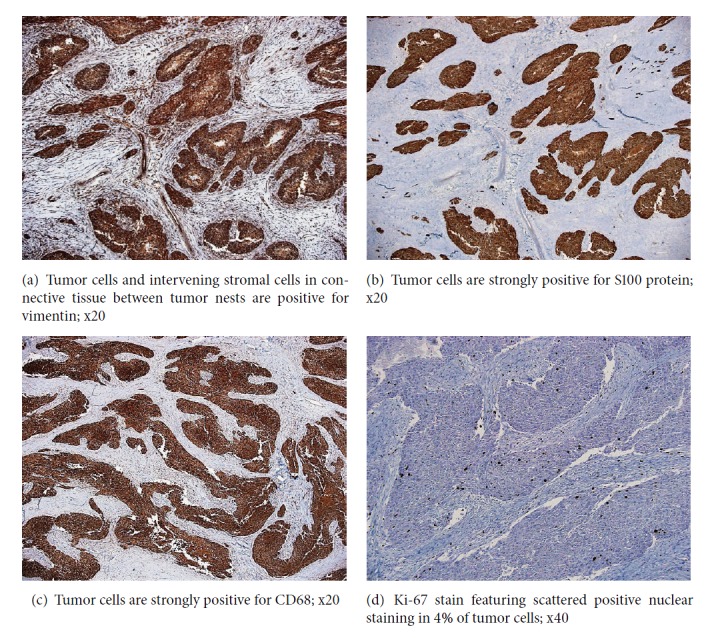


Immunohistochemical stains show the tumor cells to be strongly positive for vimentin, S100 and CD68 (Figures 2(a), 2(b), and 2(c)). Stains for cytokeratin (CK), smooth muscle actin (SMA), desmin, and myogenin were all negative. Ki-67 proliferative index was 3-5% (Figure 2(d)). PAS stain highlighted the granules and the eosinophilic globules within the cytoplasm of tumor cells.

The patient was referred in September 2007 to the Oncology Clinic at our hospital for regular follow-up. A chest CT done on May 2008 showed small right lung nodules measuring 0.48 cm of uncertain significance. In April 2010, a follow-up of her chest CT showed bilateral multiple lung nodules; the largest was in the right upper lobe measuring 1.5 x 1 cm. Pelvic CT on February 2011 revealed a large right inguinal mass measuring 3.2 x 2.1 cm. Palliative chemotherapy was planned for the patient. She was started on doxorubicin and ifosfamide for 6 cycles; the last cycle was in October 2011 and then she refused to continue treatment.

In September 2012, FNAC of the inguinal lymph node showed numerous clusters and single cells with abundant granular cytoplasm and large pleomorphic nuclei.

**Figure 3:**
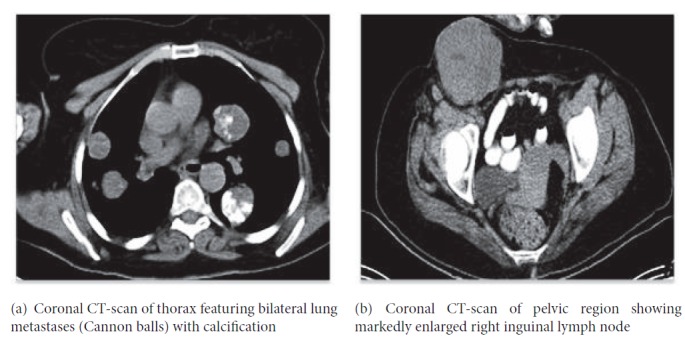


In December of 2015 she was started on docetaxel single agent for 6 cycles; the last cycle was completed by April 2016. In January 2018, her last follow-up chest CT reveled an increase in the size of all metastatic nodules. The largest nodule was in the right upper lobe and measured up to 5.2 x 4.9 cm. It had increased in size since July 2017 when it measured at that time 4.4 x 4.3 cm (Figure 3(a)). In addition, the inguinal lymph node increased in size up to 8.24 x 7.89 cm (Figure 3(b)). The patient is still alive 11 years after her initial surgery and is followed up at the Oncology Clinic.

## 3. Discussion

Alexei I Abrikossoff, a Russian pathologist, described the first case of benign GCT in the skeletal muscle of the tongue in 1926. He thought that these tumors were of skeletal muscle cells origin and called them “myoblastoma” [35]. Although the origin of these tumors is still uncertain, further research has suggested that it is mostly of Schwann cell origin [36, 37].

There are several large series of GCTs. In a study of 95 patients from Memorial Hospital in New York, with their age ranging from 11 months to 68 years, an average of 38.1 years, there were 92 benign GCTs and three MGCTs. There were 31 males and 64 females with male to female ratio of 1:2. The average size of the tumors was 1.85 cm. Multiple lesions were encountered in 8 patients. The tumor distribution was ubiquitous in many anatomic locations, including two in the abdominal wall [38]. A study of 52 GCTs in 42 patients from the Medical College of Virginia revealed an average age of 37 years, with females constituting 67% of the patients, and a dominance of the tumor in African American patients (74%). There was a high rate of multicentricity (14%). The commonest sites of involvement were the tongue (13 cases), the breast (7 cases), anogenital region (7 cases), the upper extremity (5 cases), and the abdominal wall (5 cases). These cases are reported without data on the age and sex of the patients or the size of the tumor [39]. Another clinicopathologic study of 50 cases of GCTs revealed a mean age of 38.6 years and a mean size of 2.1 cm. Most patients (64%) were females. There was a predilection for the upper extremities and the upper trunk [40].

On the other hand, MGCT is a rare and aggressive tumor, representing less than 2% of all GCT and 0.2% of all soft tissue sarcomas [3, 9]. Ordonez reviewed 43 cases of MGCTs published by 1998. There were 14 males and 29 females with male to female ratio of 1:2. The age ranged from 23 to 82 years, with a mean of 50 years. The size of the tumor ranged from 2 to 17 cm, with a mean of 7 cm. Three patients had multiple tumors. The commonest site of involvement was the lower limb (11 cases) [41]. In a review of 113 cases of MGCTs from Surveillance, Epidemiology, and End Results (SEERS) database, the median size of the tumor was 4 cm [42], unlike other benign GCTs with median size of less than 3 cm [1]. Most MGCTs were located within the soft tissues and the skin. The median age was 54 years, and 77.0% were female [42]. In a recent review of the literature of 157 reported cases of MGCT, the median tumor size was 6 cm. The median age was 51 years. There were 99 females (63% of total cases) and 58 males (37%), with female to male ratio of 1.7:1. The commonest sites of the tumor were the trunk and the thighs [11]. Fanburg-Smith et al. reviewed 46 cases of MGCTs from the Armed Forces Institute of Pathology, with a mean age of 40 years. There were 32 females, 12 males, and 2 patients with unknown age. Male to female ratio was 1:2.7. The upper extremity was the commonest site affected (40%), followed by trunk (38%) and the lower extremity (22%) [9].

Histologically, sheets of polygonal to spindly cells were shown, with an abundant granular eosinophilic cytoplasm with scattered globules; both are positive for Periodic Acid-Schiff (PAS) stain [10]. Immunohistochemical stains of all GCTs are essentially the same, which are positive for S100, vimentin, NSE, and to lesser extent for CD68 and CD57 [1].

The abdominal wall is an unusual site for benign and malignant GCT; 29 cases were reported in the literature, including our case (Table 1). Eleven cases were MGCTs, one case was atypical, and the remaining 17 were benign GCTs. There were 19 females and 10 males, with ages ranging from 6 to 70 years. The average size of benign GCT was 4.0 cm (0.5-10 cm), while MGCT averaged 7.25 cm (4.5-11 cm). Five patients with MGCT did not develop recurrence or metastatic deposits. On the other hand, six patients developed recurrences and/or metastatic deposits in distant sites including lymph nodes, lungs, liver, breast, and bone [13–34]. Not included in the table are five cases of GCTs out of 42 cases reported by Vance and Hudson due to lack of data on those cases [39].

In 1998, Fanburg-Smith et al. established the criteria for diagnosing benign, atypical, and malignant granular cell tumors based on histological features which included necrosis, spindling, vesicular nuclei with prominent nucleoli, increased mitotic activity (greater than 2 mitoses/10 HPF at a magnification of 200), high nuclear cytoplasmic ratio, and pleomorphism. MGCT was diagnosed when three or more of these features were present. Neoplasms were classified as atypical when one or two of those features were present and benign if there was only focal pleomorphism but no other features were present. Also, a tumor was considered malignant when it metastasized or caused patient death regardless of the cytological features. Metastases were the only conclusive criteria for malignancy [9]. Nasser et al. stressed the role for ki67 proliferation index in the diagnosis of GCT with potential aggressive behavior in 48 cases of GCT, including 11 cases of atypical and malignant granular cell tumors. However, these authors emphasized that metastases remained the sole definitive criterion for malignancy [43].

The present case shows at least 4 histological criteria set by Fanburg-Smith et al., namely, 4 mitosis/ 10 HPF x 200, nuclear pleomorphism, foci of necrosis, and high nuclear cytoplasmic ratio. However, the presence of subsequent metastatic deposits in both lungs and the right inguinal lymph nodes is definite feature of malignancy.

We utilized fine-needle aspiration cytology (FNAC) to establish the diagnosis of metastatic deposits of MGCT in the right inguinal lymph node. Several papers documented the importance of FNAC in diagnosing GCTs [19, 25, 44, 45]. Wieczorek et al. evaluated 3 MGCTs and 17 benign GCTs (comprising 17 fine-needle aspiration biopsy samples and 3 samples from direct scrapes) for the following cytological features: hyperchromasia; coarse chromatin; nuclear-to- cytoplasmic (N/C) ratio; nuclear pleomorphism; and vesicular nuclei with enlarged nucleoli, mitoses, necrosis, and spindle cell morphology. All these features were associated the most closely with malignancy when they were present throughout the cytologic sample [44].

The differential diagnosis of MGCT includes pleomorphic rhabdomyosarcoma, granular leiomyosarcoma, and alveolar soft part sarcoma (ASPS) [46]. MGCTs are negative for muscle markers, and rhabdomyosarcomas are almost always negative for S100 protein. In a review of nine smooth muscle tumors with granular cell changes, including four malignant ones, all tumors were S100 protein negative and were positive for anti-muscle actin antibody (HHF-35) [47]. Macarenco et al. reported a case of leiomyosarcoma of the saphenous vein with granular cell changes. They stated that other neoplasms can exhibit granular cell change including dermatofibrosarcoma protuberans, angiosarcoma, atypical fibroxanthoma, dermatofibroma, basal cell carcinoma, and melanocytic nevi. The granular cells in their cases stained negative for S100 protein, but positive for CD68. The spindle cell component of the tumor stained positive for smooth muscle actin [48]. Alveolar soft part sarcoma has a distinctive organoid pattern and lacks S100 protein positivity [46].

MGCTs have a poor prognosis with 32% local recurrence and 50% metastatic rate [9]. It can metastasize several years following the initial surgical excision, as in our case, and can recur before that. However, benign and atypical GCTs have favorable outcome with no potential for metastasis [1, 9]. Common metastatic sites for MGCT are lymph nodes, lungs, liver, and bones. It has 39% mortality rate in 3-year interval [9]. Our patient had prolonged survival although she had significant metastatic disease. It has been established that older age group, larger tumor size, local recurrence, metastasis, Ki67 >10%, and p53 immunoreactivity are all adverse prognostic factors [9].

The treatment of choice is complete surgical resection with safe margins and regional lymph node dissection. Marked resistance of the tumor to radiotherapy and chemotherapy made them of low value [8, 9]. Some authors stated that even when negative margins are not obtained, the prognosis is still favorable [9]. Follow-up guidelines for MGCTs are needed, although annual follow-up is advised to rule out local recurrence or metastatic spread [7].

## 4. Conclusion

Our patient had prolonged survival in spite of the presence of metastatic tumor deposits in both lungs and right inguinal lymph node. She received chemotherapy but it is not clear whether this contributed to her long survival or not. The presence of metastases is currently considered as the only unequivocal sign of true malignancy. This opinion is supported by some degree of overlap in clinicopathologic data between benign and malignant GCTs [1, 2]. Only a large tumor size (> 5 cm) is considered a clinical sign of potential malignancy.

## Figures and Tables

**Table 1:** Reported cases of benign, atypical, and malignant granular cell tumor of the abdominal wall.

No	Authors	Age	Gender	Size Cm.	Type	Location in Abdominal Wall	Metastasis, outcome and remarks
	Year. (Reference)						
1	Cave et al Case 3 1955 [13]	6.5	Female	2	Benign	Dermis and subcutaneous	Not applicable. Patient had one tumor in right submental area of head and one tumor in the abdominal wall.

2	Baraf and Bender Case 1 1964 [14]	29	Male	0.5-2.5	Benign	Dermis and subcutaneous	Not applicable. Patient had multiple cutaneous GCTs (21 tumors) at various sites including abdominal wall

3	Baraf and Bender Case 2 1964 [14]	18	Male	0.5-3	Benign	Dermis and subcutaneous	Not applicable. Patient had multiple cutaneous GCTs (15 tumors) at various sites, including abdominal wall.

4	Baraf and Bender Case 3 1964 [14]	33	Male	0.5-1.5	Benign	Dermis and subcutaneous	Not applicable. Patient had multiple cutaneous GCTs (about 40 tumors) within ten years at various sites

5	Gorelkin et al. 1978 [15]	58	Female	8	Benign	Intramuscular	Not applicable

6	Apisarnthanarax Case 6 1981 [16]	43	Female	3	Benign	Subcutaneous	Not applicable

7	Apisarnthanarax Case 7 1981 [16]	36	Female	2.5	Benign	Subcutaneous	Not applicable

8	Kucan et al 1982 [17]	8	Male	Small. Size not stated	Benign	Dermis and Subcutaneous	Not applicable. Patient had multiple [18] GCTs of the skin in trunk, upper and lower extremities, and buttocks

9	Geisinger et al. 1985 [19]	69	Female	Not stated	Malignant	Not stated	Metastases to lungs and lymph nodes. Dead of disease at 96 months post diagnosis.

10	Rifkin et al. Case 1 Mother 1986 [20]	23	Female	Not stated	Benign	Subcutaneous	Not applicable. Patient had total of 57 cutaneous GCTs excised during 13-year period from age 10 to 23 years at various sites including abdominal wall

11	Rifkin et al. Case 2 Son 1986 [20]	6	Male	Not stated	Benign	Subcutaneous	Not applicable. Patient had tracheal GCT with a recurrence, two abdominal wall tumors and a perianal tumor

12	Rubenstein et al Case 1 1987 [21]	7	Female	3	Benign	Subcutaneous	Not applicable. Patient had multiple [11] subcutaneous GCTs in fingers, arms, neck, buttock, abdominal wall, and the largest involved the clitoris (4.5x5.5 cm)

13	Khansur et al 1987 [22]	Not given	Male	Not stated	Malignant	Not stated	Presented with systemic metastasis in the liver and lung which rapidly progressed and caused death in five months. Son had MGCT of chest wall.

14	Vamsy et al. 1992 [23]	30	Female	10x8x5	Malignant	Intramuscular	None. Alive at 24 months postop.

15	Menaker and Sanger 1997 [24]	50	Female	4x2x2	Atypical (Uncertain Malignant Potential)	Subcutaneous	Underwent wide local excision. No recurrence or metastasis at 16 months postop.

16	Fanburg-Smith et al. Case 7 1998 [9]	32	Male	5.5	Malignant	Attached to rectus muscle sheath	No recurrences or metastasis. Alive at 2 years postop.

17	Fanburg-Smith et al. Case 17 1998 [9]	49	Female	11	Malignant	Not stated	No recurrence or metastasis. Alive at 7 years postop.

18	Joshi and Aqel 2003 [25]	37	Male	2x1.6	Benign	Intramuscular	Not applicable

19	Chelly et al. 2005 [18]	67	Female	6x4x3	Malignant	Subcutaneous and intra-muscular	None. Patient died due to pulmonary embolism three months postop.

20	An et al. 2007 [26]	44	Female	4	Benign	Intramuscular	Not applicable

21	Chaudhry et al 2008 [27]	70	Female	10	Benign	Intramuscular	Not applicable. Alive and well 5 months postop.

22	Panunzi et al. 2012 [28]	29	Female	1.5	Benign	Adherent to muscle	Not applicable

23	Chen et al. 2012 [29]	56	Female	6x3.3	Malignant	Subcutaneous with multiple recurrences	Initial abdominal wall tumor in 2003. Multiple recurrences in 2004, 2006, 2007 and 2009. Mets to right breast and axillary lymph nodes. All resected. Alive 27 months postop.

24	Toelen et al. 2013 [30]	68	Female	3	Benign	Subcutaneous	Not Applicable

25	Porta et al. 2015 [31]	45	Female	3	Benign	Intramuscular	Not Applicable

26	Liu et al. 2015 [32]	66	Male	3x2.8	Malignant	Dermis and Subcutaneous	No recurrence or metastasis at 12 months postop.

27	Yoon et al. 2016 [33]	66	Male	4.5x3.4x3	Malignant	Intramuscular	No metastasis or recurrence at 30 months.

28	Imanishi et al. Case 13 2016 [34]	48	Male	8	Malignant	Not stated	Local recurrence at 27 months. Mets to lung and bone at 26 months. Died at 87 months postop.

29	Alnashwan et al. Current case 2019	50	Female	7x6	Malignant	Subcutaneous	Metastases to both lungs and right inguinal lymph node. Alive at 132 months postop.
